# Mesoporous Starch Cryoaerogel Material as an Emerging Platform for Oral Drug Delivery: Synthesis and In Vitro Evaluation

**DOI:** 10.3390/gels9080623

**Published:** 2023-08-02

**Authors:** Samira Jafari, Farzaneh Khodaensaf, Cédric Delattre, Vahid Bazargan, Paolina Lukova

**Affiliations:** 1 Pharmaceutical Sciences Research Center, Health Institute, Kermanshah University of Medical Sciences, Kermanshah 6714415153, Iran; f.khodaensaf1996@gmail.com; 2 School of Mechanical Engineering, College of Engineering, University of Tehran, Tehran 1439957131, Iran; 3 Institut Universitaire de France (IUF), 1 rue Descartes, 75005 Paris, France; cedric.delattre@uca.fr; 4 Clermont Auvergne INP, CNRS, Institut Pascal, Université Clermont Auvergne, 63000 Clermont-Ferrand, France; 5 Department of Pharmacognosy and Pharmaceutical Chemistry, Faculty of Pharmacy, Medical University-Plovdiv, 4002 Plovdiv, Bulgaria

**Keywords:** cryoaerogel, starch, mesoporous, atorvastatin, drug delivery

## Abstract

In this study, a starch cryoaerogel formulation was developed as a carrier for poorly water-soluble drugs, like atorvastatin. Cryoaerogels were generated through a sol–gel method combined with a freeze-drying technique, and atorvastatin was incorporated into the obtained mesoporous systems during the solvent exchange stage. The formulated drug-loaded polymer structures were characterized in terms of their physicochemical properties, solid-state behavior, and cytotoxicity. They had a pore size of 27.56 nm and a drug loading size of 38.60%. Fourier transform infrared (FTIR) and scanning electron microscopy (SEM) analyses indicated that atorvastatin was successfully incorporated into the cryoaerogel pores. The amorphous nature of the loaded drug was confirmed via X-ray diffraction (XRD). Furthermore, after the atorvastatin incorporation into the cryogel, the volume of nitrogen adsorbed on one gram of cryoaerogel (Vm), as well as the specific surface area (aBET) were reduced. The comparison between the drug release profiles of crystalline atorvastatin and the loaded formulation of atorvastatin showed that by including the drug into the pores of the developed cryoaerogel matrix its solubility was significantly improved—the time for the dissolution of 30% pure atorvastatin (t_30%_) was approximately 4 h, whereas the determined t_30%_ for the formulated cryoaerogels was only 1 h. Moreover, the data from the MTT assay illustrated that the designed cryoaerogel could be used as a safe oral atorvastatin delivery system. According to obtained results, it could be concluded that the starch cryoaerogel formulation is a promising candidate for oral delivery of poorly water-soluble therapeutic agents.

## 1. Introduction

Atorvastatin calcium (ATV), as a lipid-lowering agent, is administered to manage hypercholesterolemia- and dyslipidemia-related disorders. It is beneficial to inhibit cardiovascular disease, and consequently, it is able to attenuate myocardial infarction risk, as well. This therapeutic agent belongs to a class II drug based on the biopharmaceutical classification system (BCS), which is insoluble in aqueous solutions [1]. Issues, like poor oral bioavailability, poor aqueous solubility, hepatic first-pass metabolism, and so forth, lead to restrictions in its clinical profits. To address these challenges, various strategies have been assessed in the literature [2,3,4,5]. The designing of mesoporous structures, as drug delivery systems, has been highlighted by many authors as a successful approach to enhance drug bioavailability and improve drug therapeutic efficacy.

Aerogels are a group of lightweight porous materials with attractive attributes, like high specific surface area, large open pores, low refractive index, and low density, which make them a promising candidate for a wide variety of applications, like thermal insulation, food industry, energy storage devices, environmental cleanup, biomedical applications, etc. [6,7]. These porous structures are most commonly fabricated through sol–gel techniques using molecular inorganic/organic precursors and subsequent elimination of the solvents from the wet gels via appropriate drying, like freeze-drying, etc. [8,9]. Gelation temperature, the type of the precursor used in the sol, the retrogradation time and temperature, the solvent exchange duration, and the drying method are some of the key factors, which should be considered in the preparation of such drug delivery systems. Aerogel materials can be produced from a hydrogel, applying an appropriate drying technique for water displacement. If gels are prepared following a straightforward freeze-drying method, cryoaerogel structures can be formulated. Due to the freezing of the gel and the subsequent sublimation to remove the solvent, a highly porous solid structure is obtained [10,11,12,13,14,15,16,17].

A wide variety of compounds, including polymers, metal oxides, carbon structures, proteins, and polysaccharides can be employed to generate such gel formulations. In recent years, natural polymer-based, particularly polysaccharide-based, gels have received tremendous interest by researchers. These biopolymers in comparison with inorganic precursors materials possess unique properties, such as biodegradability, biosafety, biocompatibility, and availability, which are essential characteristics for biomedical applications. Alginate, chitin, chitosan, pectin, and starch have been reported as prominent polysaccharides to formulate such gel structures. Furthermore, the free hydroxyl groups in these biomaterials are beneficial for their functionalization and efficient applicability as pharmaceutical formulations. Among the various polysaccharides, starch has received much attention, particularly for the development of cryoaerogels for biomedical applications. This natural polymer is safe, nontoxic, non-allergenic, inexpensive, and also abundant [13]. In a recent study, a mesoporous tablet-shaped potato starch aerogel was developed as carriers of celecoxib in order to enhance the drug solubility. The obtained data revealed that the prepared aerogels were able to improve the dissolution rate of the loaded celecoxib compared to the pure drug [14]. In another research study, using the Minitab experimental design software, the major factors for the aerogel synthesis along with optimal values of these parameters were specified. The formulated aerogels were consequently loaded with ibuprofen as a model drug, and drug release was investigated. Based on the release profiles reported in this study, the ibuprofen loaded in the aerogels dissolved faster than crystalline ibuprofen [15]. In a research report, a nanocellulose/gelatin composite of cryoaerogels with controllable porosity were prepared to deliver 5-fluorouracil. The drug-loaded cryoaerogels demonstrated sustained in vitro drug release, which was related to the hydration of gelatin as well as the reversible hydrolysis of acetal/hemiacetal. The drug release profiles were dependent on the density of the formulated structures, the degree of their cross-linking as well as the pH. The cryogel carriers showed sustained release up to 12 h in a simulated intestinal environment [16].

In addition, in a similar research, Bajpai et al. reported the formulation of a ciprofloxacin-loaded poly(vinyl alcohol) cryogel without using cross-linking agents. It was found that the drug release profile was considerably influenced by the drug loading, the composition of the cryogel, pH, and temperature. The increase in the drug loading in the cryoaerogels led to the increase in the drug release while the number of freeze–thaw cycles had a negative impact on the drug release; an increase in the number of freeze–thaw cycles led to a decrease in the released amount of the drug. Moreover, the lowest release was observed in physiological pH (7.4), whereas the optimum release was reported in pH 8.0. Additionally, a rise in temperature up to 40 °C provided an increase in the released ciprofloxacin [17].

In the present study, starch has been blended with poly(vinyl alcohol) to design macroporous architectures following a repeated freeze–thaw method. These macroporous cryoaerogels were loaded with an antibiotic drug, ciprofloxacin hydrochloride (Cfx), and evaluated for its in vitro delivery in a completely controlled manner, thus exploring possibilities to use it as a biomaterial in burn- or wound-healing applications. The key advantage of the present system is that cryoaerogels formed do not contain any chemical crosslinking agent, which is often harmful to organic compounds. These Cfx-loaded cryoaerogels were characterized by infrared (FTIR) spectroscopy and scanning electron microscopy (SEM) techniques. The controlled release of Cfx drug from cryoaerogels was investigated under varying experimental conditions, such as percent-loading of the antibiotic drug, chemical architecture of the cryoaerogels and pH, temperature, and the nature of the release media. The prepared cryoaerogels show promise to provide a possible pathway for controlling the delivery of antibiotic drugs, thus minimizing the known side effects and improving efficacy also.

Therefore, the aim of the present study was to develop starch cryoaerogels with a hierarchical and an interconnected pore structure as a plausible approach for formulating without organic solvents a drug delivery system for atorvastatin with an improved drug dissolution profile. To accomplish the proposed strategy, cryoaerogels were prepared through a sol–gel technique followed by a freeze-drying method, and the enhancing effect of the obtained formulation on the ATV dissolution was evaluated.

## 2. Results and Discussion

A suitable formulation of a starch cryoaerogel was prepared using the sol–gel method, combined with a freeze-drying technique. In order to use the developed polymer structure as a delivery system for atorvastatin, the model drug was incorporated into the pores of the formulated mesoporous starch cryoaerogel. The proposed formulation was characterized in terms of surface morphology using SEM, compatibility between the drug and the polymer using FTIR, solid state of the drug via XRD analysis, surface area, and adsorption isotherms. Finally, since the main aim of this study was to develop a drug delivery system with improved atorvastatin dissolution, the designed formulation was assessed for its in vitro drug release profile and cytotoxicity. 

### 2.1. Drug Loading

Atorvastatin was incorporated in the pores of the synthesized starch cryoaerogel by a batch adsorption method during the final stage of the solvent substitution process. According to the obtained results, the formulation containing 800 mg of ATV had the highest drug loading value (38.60%) (Table 1).

### 2.2. SEM Analysis

**Figure 1:**
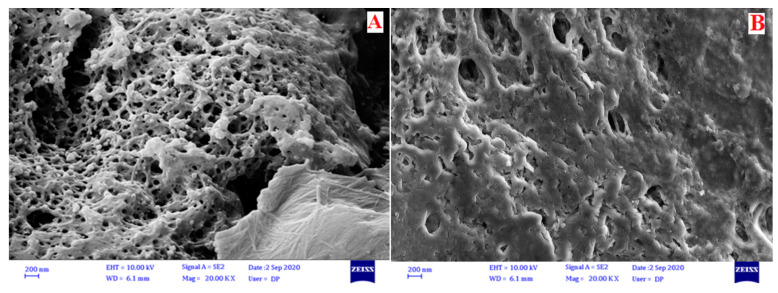
SEM images of (**A**) a drug-free cryoaerogel and (**B**) an ATV-loaded cryoaerogel (20,000×).

SEM images of the drug-free and ATV-loaded cryoaerogels are presented in Figure 1A,B, respectively. As shown, the drug-free cryoaerogels had a porous structure composed of spherical particles, while the surface morphology and particularly the porosity of the obtained systems were altered due to the loading of the drug in the pores of the synthesized cryoaerogels. Similar morphology has previously reported by Bölgen for starch cryogels [18].

### 2.3. Fourier Transform Infrared Spectroscopy (FTIR)

**Figure 2:**
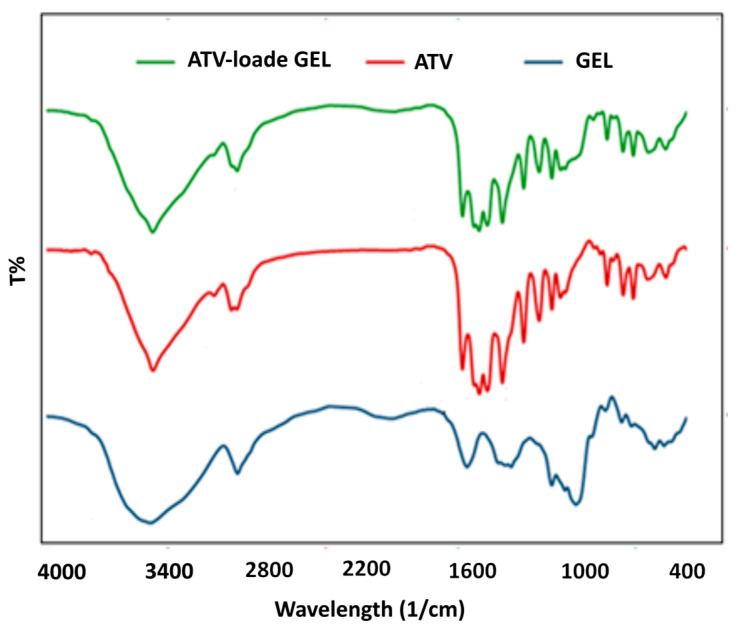
FTIR spectra of the cryoaerogel, ATV, and ATV-loaded cryoaerogel.

The recorded FTIR peaks in Figure 2 could be used not only to identify the functional groups of the drug and the developed cryoaerogel but also to estimate possible interactions between the drug and the carrier. In the cryoaerogel spectrum, distinctive absorption peaks were observed at 3414 cm^−1^ (O–H stretching) and 2927 cm^−1^ (=CH stretching), 1631 cm^−1^ (C=O), 1454 cm^−1^ (C=C), and 1022 cm^−1^ (C-O-C). The ATV spectrum demonstrated characteristic peaks at 3402 cm^−1^ (N-H stretching), 2931 cm^−1^ (C-H aromatic stretching vibrations), 1658 cm^−1^ (C-H aliphatic stretching vibrations), 1435 cm^−1^ (C=O symmetric and asymmetric stretching), and 1315 cm^−1^ (C-C), 1226 cm^−1^ (C-N aromatic stretching vibrations). The comparison between the three recorded spectra (atorvastatin, drug-free, and ATV-loaded cryoaerogel) indicated the absence of chemical interactions between ATV molecules and the cryoaerogel matrix. Similar results were established for starch-based cryoaerogels prepared via sol–gel and freeze-drying methods [17].

### 2.4. XRD Analysis

**Figure 3:**
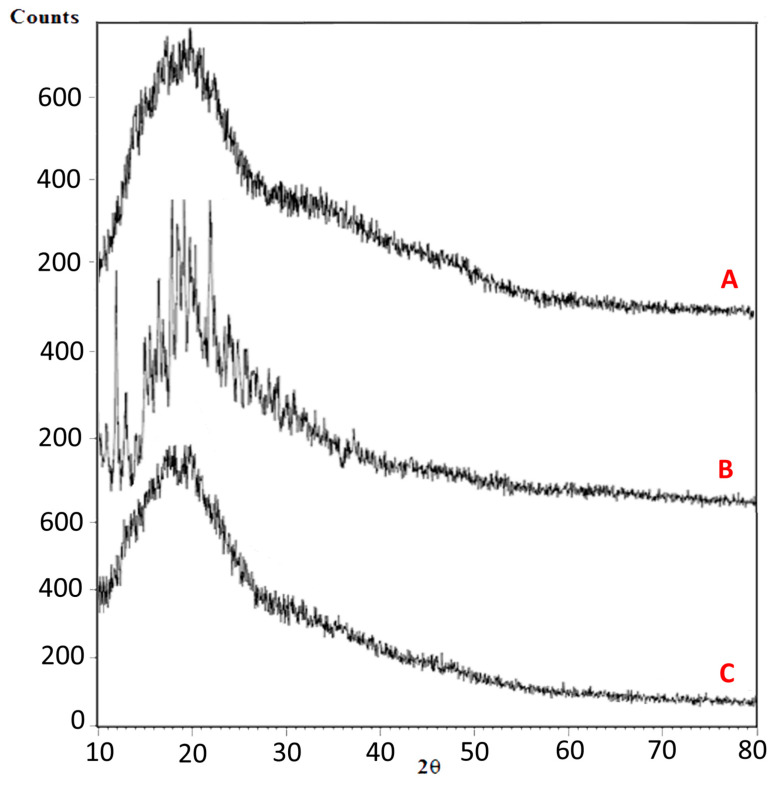
XRD patterns of (**A**) cryoaerogel, (**B**) ATV, and (**C**) ATV-loaded cryoaerogel.

The plausible changes in the ATV structure during the drug loading process, was evaluated via an XRD analysis. According to the XRD diagram (Figure 3), the crystalline state of the drug structure of atorvastatin leads to numerous and intense diffraction peaks that are visible in the spectrum, which are related to the tetragonal and monoclinic structure of atorvastatin. The broad peak around 18° indicates the non-crystalline and amorphous structure of the synthesized cryoaerogel, which did not shift after the addition of the drug atorvastatin, and the peaks related to the drug disappeared. In fact, the process of forming an aerogel has led to the loss of the granular crystalline structure of starch and its transformation into an amorphous cryoaerogel structure. As it is clear in Figure 3, new peaks were not formed, but the intensity of the cryoaerogel peaks increased, which indicated the successful loading of atorvastatin. The absence of characteristic drug peaks confirms that the ATV drug loaded in the pores is in a non-crystalline state. The lack of crystallization may be attributed to the hydrogen bonding in the cryoaerogel pores with the drug, as well as to the encapsulation of the drug inside the cryoaerogel pores. Recently, similar results were presented by Reverchon et al. for the starch aerogel loaded with poorly water-soluble vitamins [19].

### 2.5. Surface Area Determination

**Figure 4:**
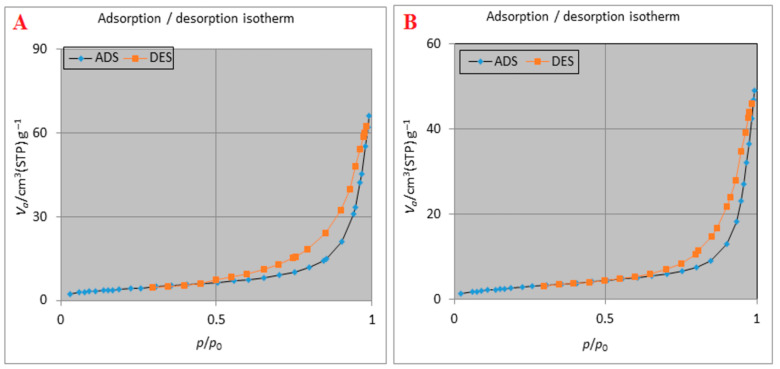
Nitrogen adsorption/desorption isotherms of cryoaerogel before (**A**) and after ATV loading (**B**).

The obtained data from BET analysis included the specific surface area along with porosity attributes—the volume of nitrogen adsorbed on a gram of the cryoaerogels to complete the monolayer adsorption (V_m_) of the prepared samples were summarized in Table 2. According to the recorded data, all the parameters were reduced after the drug loading process due to the inclusion of the drug molecules into the structure of the mesoporous cryoaerogels. Furthermore, the isotherm of nitrogen adsorption–desorption related to the drug-free cryoaerogels as well as ATV-loaded cryoaerogels are presented in Figure 4. It was observed that at a relative pressure above 0.5 and 0.7, respectively, for drug-free cryoaerogels and ATV-loaded cryoaerogels samples, plenty of porous adsorbents desorbed more vapor rather than the corresponding amount of absorbed vapor. This phenomenon subsequently resulted in a generation of hysteresis loops in the adsorption–desorption isotherms. Access to data on the geometric shape of mesopores with capillary condensation was achieved by hysteresis loops. In agreement with IUPAC classification, the IV-type isotherm with H_3_-type hysteresis was observed for both samples. The IV-type isotherm and H_3_-type hysteresis loops can be attributed to capillary condensation and the asymmetric slot shape of mesopores, respectively, in the adsorbent structure [20]. Based on the data in Table 2, there was only a slight change in the pore size before (25.99 nm) and after drug loading (27.57 nm), which may indicate that the drug retained could not completely block the mesoporous entrances. Additionally, it is noteworthy that the pore size of the prepared cryoaerogels belongs to the mesoporous materials (2–50 nm) [21,22].

### 2.6. Adsorption Isotherms

**Figure 5:**
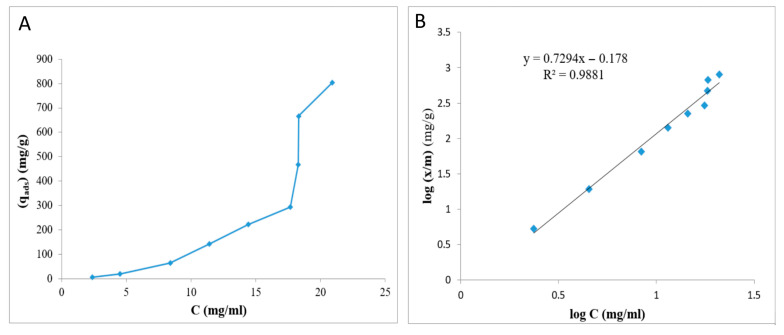
Non-linear (**A**) and linear (**B**) plots of the Freundlich isotherm.

The equilibrium adsorption isotherms (Langmuir and Freundlich isotherms) were used to obtain information about the adsorption mechanisms, the surface properties of the cryoaerogels and their affinity to the adsorbent [22]. Generally, the Langmuir and Freundlich isotherms are related to a monolayer adsorption on the uniform homogeneous and multilayer adsorption on the heterogeneous surface of the adsorbent, respectively, [23]. In the present analysis, the adsorption equilibrium data were adjusted to the Freundlich isotherm. The provided equation is associated to the linear form of the Langmuir isotherm [24]:

$$lnq_{e}=lnk_{F}+\left(\frac{1}{n}\right)lnC_{e}\;$$

where $q_{e}$ is the adsorbed drug amount (mg/g); $C_{e}$ is the equilibrium concentration of the adsorbate (mg/mL); $k_{F}$ (adsorption capacity), and $\frac{1}{n}$ (surface heterogeneity) is the Freundlich constant, which determine deviation from adsorption linearity. These parameters could be characterized through slope and intercept of the presented linear plot in Figure 5B. Normal adsorption is observed when n < 1; n = 1 indicates independency of the adsorption process from the concentration; and n > 1 displays a cooperative adsorption [25].

### 2.7. Thermogravimetric Analysis

**Figure 6:**
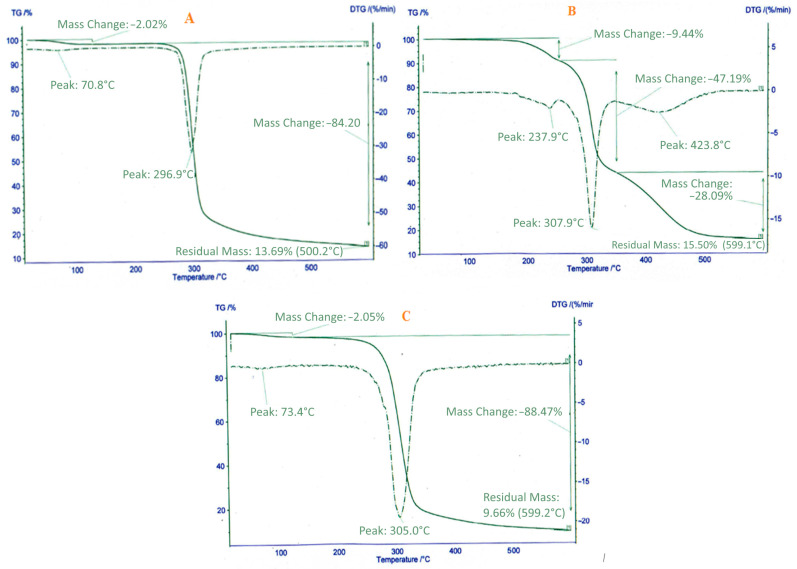
TGA results of (**A**) the synthesized cryoaerogel, (**B**) atorvastatin, and (**C**) drug loaded in the cryoaerogel system at 23 °C temperature and 53% humidity.

TGA analysis was used to determine the percentage of organic functional groups loaded in the cryoaerogel structure. In Figure 6, thermograms A, B, and C showed the cryoaerogel, atorvastatin drug, and the aerogel containing atorvastatin, respectively. In both the thermograms obtained from the cryoaerogel and the cryoaerogel containing the drug atorvastatin, the two percent weight loss at a temperature of about 70 °C can be attributed to the physically absorbed water in the cryoaerogel cavities, and the weight loss above 150 °C was due to the decomposition and destruction of the organic parts of the structure. Cryoaerogel was included, which is well evident in Figure 6A,C at a temperature of about 300 °C with a decrease of 84.20 and 88.47%, respectively. The thermogram related to the atorvastatin contained three peaks at a temperature above 150 °C, and the main decomposition and destruction of the drug occurred at a temperature of around 307 °C with a weight loss of about 48%. It is interesting to note that after loading the drug in the aerogel structure, the percentage of weight loss at 300 degrees was increased by 4%, which can be related to the drug. These results proved that the drug was successfully loading in the aerogel structure.

### 2.8. In Vitro Drug Release

**Figure 7:**
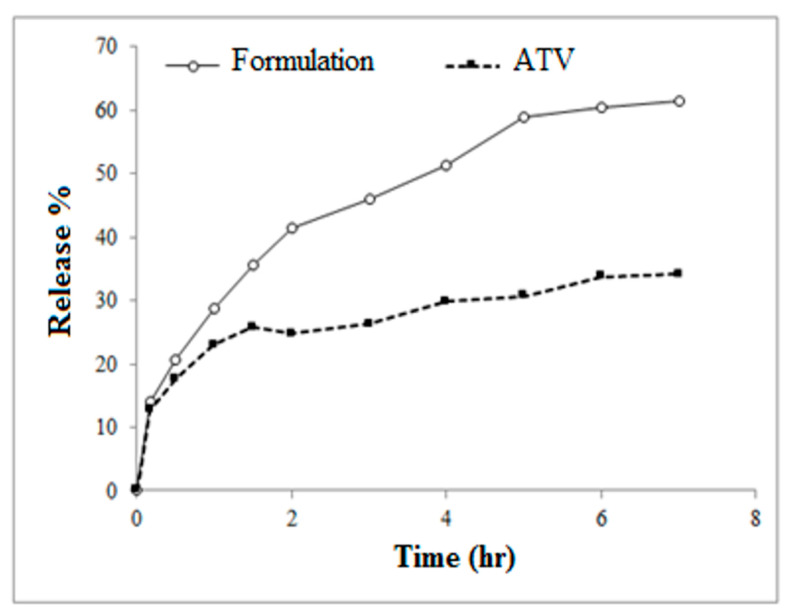
In vitro drug release profiles of pure ATV and ATV-loaded cryoaerogel formulation.

The in vitro release profiles of the pure drug and the drug loaded in the cryoaerogel structure are presented in Figure 7.

As shown, a significant difference between the release profiles of pure ATV and ATV from the formulated cryoaerogel was observed. The time for the dissolution of 30% pure atorvastatin (t_30%_) was approximately 4 h, whereas the determined t_30%_ for the formulated cryoaerogels was only 1 h. Furthermore, Q_7h_ (the percent of the drug dissolved within 7 h) was 33% and 60% for the pure drug and the cryoaerogel, respectively. The comparison between the obtained release patterns of ATV and ATV from the cryoaerogel clearly indicated that after inclusion into the cryoaerogel mesoporous structure of the drug showed an enhanced/accelerated dissolution rate, which could be attributed to the amorphous form of atorvastatin in the prepared formulation. In general, the amorphous structure of a drug is associated with higher dissolution rates compared to its crystalline form. Additionally, according to the literature, hydrophilicity and the interconnections in the mesoporous platforms also play an important role in the drug dissolution rate. These characteristics of the matrix are essential for the penetration of the dissolution media into the pores of the cryoaerogel, the destruction of its network structure, and the subsequent drug release [26,27,28].

### 2.9. Cytotoxicity Assessment

**Figure 8:**
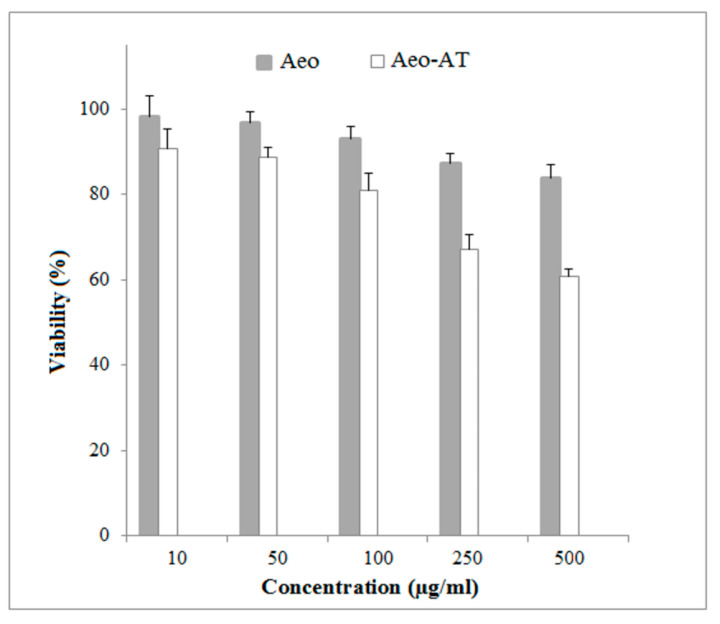
Cell viability of NIH-3T3 cell line after 24 h in response to different concentrations of drug-free cryoaerogel (Aeo) and atorvastatin-loaded cryoaerogel (Aeo-AT).

The viability of NIH-3T3 upon treatment of different concentrations of ATV-free and ATV-loaded cryoaerogels after 24 h is presented in Figure 8. A dose-dependent cytotoxicity was observed for the investigated samples due to their direct interaction with the cell surfaces. As expected, ATV-loaded cryoaerogels showed a higher growth inhibitory activity on the tested cell lines in comparison with the drug-free cryoaerogels. However, the high viability of the cells treated with the developed starch cryoaerogels confirmed that the proposed formulations had the potential to be used as safe drug carriers.

## 3. Conclusions

The current study demonstrated the development of a suitable polymer cryoaerogel drug formulation. The proposed preparation method of a sol–gel technique with subsequent freeze-drying was established as an effective approach for obtaining drug-free and drug-loaded starch systems with a porous structure. According to the obtained results from FTIR, X-ray, and TGA analyses, the model drug used, atorvastatin, can be successfully incorporated into the pores of such a formulation. In agreement with the obtained release profile, the proposed drug delivery system significantly increased the solubility of the drug, providing it with amorphous properties. Q_7h_ (the percent of the drug dissolved within 7 h) was 33% for the pure drug while this parameter was 60% for the drug-loaded cryoaerogel. Inclusion of the drug into the cryoaerogel mesoporous structure as well as the amorphous form of atorvastatin in the cryoaerogel could be the two major reasons of this phenomenon. Furthermore, the biocompatibility, biodegradability of the polysaccharide used, as well as the satisfactory results from the MTT test indicated that the formulated cryoaerogel had the potential of a safe carrier for oral atorvastatin delivery. As a whole, it can be concluded that starch cryoaerogels are perspective candidates for oral delivery of the poorly water-soluble drugs.

## 4. Materials and Methods

### 4.1. Materials

Atorvastatin was purchased from Darou pakhsh Co. (Tehran, Iran). Starch, ethanol (98%), and (4.5-dimethylthiazol-2-yl)2.5-diphenyltetrazolium bromide (MTT) were received from MERCK KGaA (Darmstadt, Germany). The studied cell line (NIH-3T3) was supplied by the National Cell Bank, Pasteur Institute (Tehran, Iran). All reagents purchased were of analytical grade.

### 4.2. Starch Cryoaerogel Preparation

Starch cryoaerogels were prepared using a sol–gel technique. Briefly, three aqueous solutions of potato starch were prepared with concentrations of 5, 10, and 15% w/v by mixing the polysaccharide with distilled water. Vigorous stirring at temperature of 70 °C was applied to create a viscous milky solution. Then, the produced gels were poured into polyethylene molds (with a depth of 10 mm and a diameter of 16 mm). In the next step, to protect the porous structure of the molded gels as well as to exclude their shrinkage during drying, an exchange of water content of the gel pores with organic solvent, like ethanol, was carried out. It should be noted that the water present in the gel pores can lead to shrinking the gel and also prolonging the drying process. To replace the water, the prepared gels were immersed in ethanol 30%, 50%, 70%, and pure ethanol, respectively, on consecutive days; at this step, the molded gels were called alcogel. Finally, freeze-drying was used to remove the ethanol and to complete the cryoaerogel generation process. 

### 4.3. Cryoaerogel Drug Loading

The pores of the developed cryoaerogels were loaded with atorvastatin using a batch adsorption approach during the final stage of the solvent replacement process. To specify the optimum ATV loading capacity, tablet-shaped alcogels were placed in a solution (20 mL) containing different amounts of ATV (from 100 to 800 mg) for 48 h in closed conical bottles to prevent solution evaporation. The ATV-loaded cryoaerogels were acquired through freeze-drying of the alcogels. The prepared cryoaerogels were crushed, dispersed into 40 mL of pure ethanol at sink conditions, and then sonicated for 20 min. They were filtered through 0.22 µm syringe filters samples and were examined in order to determine the drug loading using a UV–Vis spectrophotometer at 247 nm (Shimadzu, Kyoto, Japan).

### 4.4. Scanning Electron Microscopy (SEM)

Evaluation of the surface morphology of the synthesized cryoaerogels was performed by scanning electron microscopy (SEM, MIRA3, TESCAN, USA) at 15 kV at magnifications of 10,000× and 20,000×. The cryoaerogels were mounted on aluminum stubs and coated with a thin layer of gold using a sputtering apparatus prior to investigation.

### 4.5. Fourier Transform Infrared Spectroscopy (FTIR)

FTIR spectroscopy (Model Shimadzu 43000, Shimadzu Corporation, Kyoto, Japan) was conducted to identify the functional group as well as the chemical bonds in the starch cryoaerogels, ATV, and drug-loaded cryoaerogels. The recorded spectra were investigated in the range from 4000 to 400 cm^−1^ with a resolution of 2 cm^−1^.

### 4.6. X-ray Diffraction (XRD)

An X-ray diffractometer (Model D5000, Siemens AG, Munich, Germany) under Cu kα radiation (λ of 1.5405°) was used to analyze the crystallinity structure of the pure drug, the drug-free cryoaerogel, and the drug-loaded cryoaerogel. The diffraction graphs were scanned in the range of 10 to 80 with a rate of 0.06° min^−1^.

### 4.7. BET Analysis

Nitrogen adsorption/desorption was carried out using Belsorp mini II analyzer (MicrotracBEL, Osaka, Japan) to evaluate the surface area, total pure volume, and pore size of the prepared samples at a constant temperature (77 k) at a continuous adsorption condition. Brunauer–Emmett–Teller (BET) and Barrett–Joyner–Halenda (BJH) analyses were applied to determine specific surface area and pore size, respectively.

### 4.8. Thermogravimetric Analysis

A thermogravimetric analysis (TGA) of the prepared samples was performed using a thermogravimetric (TG) analyzer (Model Q600, TA Instruments, New Castle, DE, USA). All samples were analyzed under a nitrogen atmosphere under a flow rate of 100 mL/min and a heating rate of 20 °C/min with a temperature range of 50–700 °C.

### 4.9. In Vitro Drug Release

The in vitro release of ATV from the optimum ATV-loaded cryoaerogels was conducted using s USP apparatus II, a paddle stirrer model (Erweka dissolution tester) at a paddle speed of 50 rpm, and a temperature of 37 °C. The release profile was evaluated in a 0.1 M phosphate buffer saline—simulated intestinal fluid, pH 7.4. The prepared tablets of the ATV-loaded cryoaerogel were immersed in 50 mL dissolution medium. At predetermined time intervals (0.25–8 h), 3 mL aliquot was withdrawn and filtered via a cellulose acetate membrane with the pore diameter of 20 nm. The withdrawn volume was immediately replaced by 3 mL of fresh buffer. To measure ATV content, all the samples were assessed at a wavelength of 247 nm using a UV spectrophotometer, and then the cumulative ATV release was plotted against time.

### 4.10. Cytotoxicity Assay

To evaluate the cytotoxicity effects of pure ATV as well as ATV-loaded cryoaerogels, MTT assay was performed on the fibroblast cell line (NIH-3T3). This selected cell line was cultured in a RPMI 1640 medium accompanied with 10% FBS, penicillin (10 U/mL), and streptomycin (µg/µL) on a 96-well plate in an incubator with 5% CO_2_ at 37 °C. After 24 h incubation, the cultivated cells were exposed to different concentrations of the samples (10–500 µg/mL). The replacement of the culture medium was performed with 150 μL fresh media plus 50 μL MTT reagent (2 mg/mL in PBS) after 24 and 48 h. After that the plates were incubated for a further 4 h, the MTT solutions were substituted with Sorenson’s glycine buffer (25 µL) and DMSO (200 µL). Finally, using a spectrophotometric microplate reader (Biotek Company, Santa Clara, CA, USA), the optical density (OD) of the wells was determined.

## Figures and Tables

**Table 1:** Drug loading (DL %) values for formulations, obtained by using different cryoaerogel: atorvastatin ratios.

Formulation	Cryoaerogel (mg)	ATV (mg)	DL % ± SD
1	500	100	0.60 ± 0.27
2	500	200	3.66 ± 0.52
3	500	300	6.97 ± 0.61
4	500	400	15.17 ± 1.17
5	500	500	17.59 ± 2.45
6	500	600	25.53 ± 1.71
7	500	700	35.72 ± 0.64
8	500	800	38.60 ± 3.49

**Table 2:** Surface attribution of the cryoaerogel before (AEO) and after drug loading (ATV/AEO).

Samples	W_DH_ (nm)	S_BET_ (m^2^/g)	V_t_ (cm^3^/g)
AEO	27.565	10.723	0.07
ATV/AEO	25.997	15.394	0.10

## Data Availability

Not applicable.
